# Audiovisual crossmodal cuing effects in front and rear space

**DOI:** 10.3389/fpsyg.2015.01086

**Published:** 2015-07-30

**Authors:** Jae Lee, Charles Spence

**Affiliations:** Crossmodal Research Laboratory, Department of Experimental Psychology, University of OxfordOxford, UK

**Keywords:** attention, auditory perception, orientation, rear space, spatial cuing

## Abstract

The participants in the present study had to make speeded elevation discrimination responses to visual targets presented to the left or right of central fixation following the presentation of a task-irrelevant auditory cue on either the same or opposite side. In Experiment 1, the cues were presented from in front of the participants (from the same azimuthal positions as the visual targets). A standard crossmodal exogenous spatial cuing effect was observed, with participants responding significantly faster in the elevation discrimination task to visual targets when both the auditory cues and the visual targets were presented on the same side. Experiment 2 replicated the exogenous spatial cuing effect for frontal visual targets following both front and rear auditory cues. The results of Experiment 3 demonstrated that the participants had little difficulty in correctly discriminating the location from which the sounds were presented. Thus, taken together, the results of the three experiments reported here demonstrate that the exact co-location of auditory cues and visual targets is not necessary to attract spatial attention. Implications of these results for the design of real-world warning signals are discussed.

## Introduction

Our senses are constantly bombarded by information from the surroundings, and therefore it is crucial for our brains to know which stimuli should be focused on, and which can safely be ignored. Over the last few decades, there has been a plethora of research on the topic of spatial attention, spanning all the way from basic (see Spence and Driver, 2004, for a review) through to applied (see Spence and Ho, 2008, for a review). The majority of the research on this topic has been focused on exogenous (involuntary) orienting rather than endogenous (voluntary) orienting (see Spence and Driver, 2004; Wright and Ward, 2008, for reviews). In the case of endogenous spatial orienting, attention is thought to be “pushed” to the expected target location (e.g., following the presentation of an informative central arrow cue at fixation), whereas in the case of exogenous orienting, attention is “pulled” to the location of a salient peripheral cue (Spence and Driver, 1994, 2004; Wright and Ward, 2008). While early exogenous cuing studies tended to focus on spatial attention within just the visual modality (Jonides, 1981; Briand and Klein, 1987; Müller and Rabbitt, 1989; Rafal et al., 1991; Klein et al., 1992), there has been an explosion of research interest in crossmodal attention over the last couple of decades (e.g., Spence and Driver, 1994, 1997; McDonald et al., 2000; Ferlazzo et al., 2002).

The major finding to have emerged from these studies of exogenous spatial orienting is that participants typically respond more rapidly to targets when they are preceded by cues presented on the same side than when the cues and targets are presented on opposite sides. The facilitation attributable to exogenous spatial cuing typically lasts for around 300 ms from the onset of the cue^1^. It is, however, not clear exactly how spatially specific exogenous spatial cuing effects are: is the exact co-location of cues and targets required, or is the comparative lateral position between cues and targets all that matters, as the terms *location, position*, and/or *side* have been used interchangeably when discussing cuing effects (Posner, 1980; Posner et al., 1980; Jonides, 1981; Briand and Klein, 1987; Rizzolatti et al., 1987; Spence and Driver, 1994; Ward, 1994; Spence et al., 1998; Rorden and Driver, 1999; McDonald et al., 2000; Schmitt et al., 2000; Kennett et al., 2001, 2002; Ferlazzo et al., 2002; Batson et al., 2011).

Here, we report three experiments designed to investigate how the location of auditory cues, in terms of lateral cuing (i.e., cued if the cues and targets are on the same side and uncued if they appear on opposite sides) and depth (i.e., front vs. rear), affects the cuing effect for frontal visual targets. Experiment 1 was conducted in order to replicate the standard crossmodal exogenous auditory spatial cuing effect in front space, before going on to study what happens in rear space (Experiment 2). Both experiments adapted the orthogonal spatial cuing methodology originally introduced by Spence and Driver (1994, 2004) in which the dimension of cuing is orthogonal to that of participants’ responses. For example, if the cues happened to be presented on the z-axis (i.e., front or rear), the task in the orthogonal cuing design was to indicate whether the targets appeared on either the left or right side (*x*-axis), or on the upper vs. lower location (*y*-axis). The orthogonal cuing design allows researchers to rule out any observed performance benefits that might result simply from response priming (Spence and Driver, 1994). Spatial cuing effects were evaluated by looking for any performance discrepancy in the reaction times (RTs) and error rates (ERs) of participants’ responses between cued and uncued trials. Since a task-irrelevant auditory cue varies in a spatial dimension orthogonal to that in which target discrimination judgments are made, any cuing effect will be reflected by shorter RTs at the cued as compared to the uncued locations if the cue facilitates target perception.

## Experiment 1

Experiment 1 was designed to test the hypothesis that participants would respond significantly faster (and possibly also more accurately) to visual targets that had been preceded by cues from the same side of central fixation as compared to those presented on the opposite side. We also assessed any differences in the magnitude of the spatial cuing effects as a function of the type of auditory cue that was presented: white noise vs. pure tones. Given that white noise stimuli are easier to localize than pure tones, especially in terms of their elevation (e.g., Stevens and Newman, 1936; Deatherage, 1972; Spence and Driver, 1994), it seemed plausible to assess whether the latter might lead to a broader spread of spatial attention around the cued location.

### Methods

#### Participants

Twenty participants (10 male and 10 female) were recruited to take part in the experiment through the Crossmodal Research Lab mailing list and Oxford Psychology Research participant recruitment scheme. The average age of the participants was 26 years, with a range from 19 to 37. All of the participants were right-handed, and had normal hearing and vision, by self-report. The experimental session lasted for approximately 30 min. The participants were paid £5 in return for taking part in the study. The experiment was approved by the Medical Sciences Interdivisional Research Ethics Committee at the University of Oxford, and was conducted in line with the guidelines provided.

#### Apparatus and Materials

**FIGURE 1:**
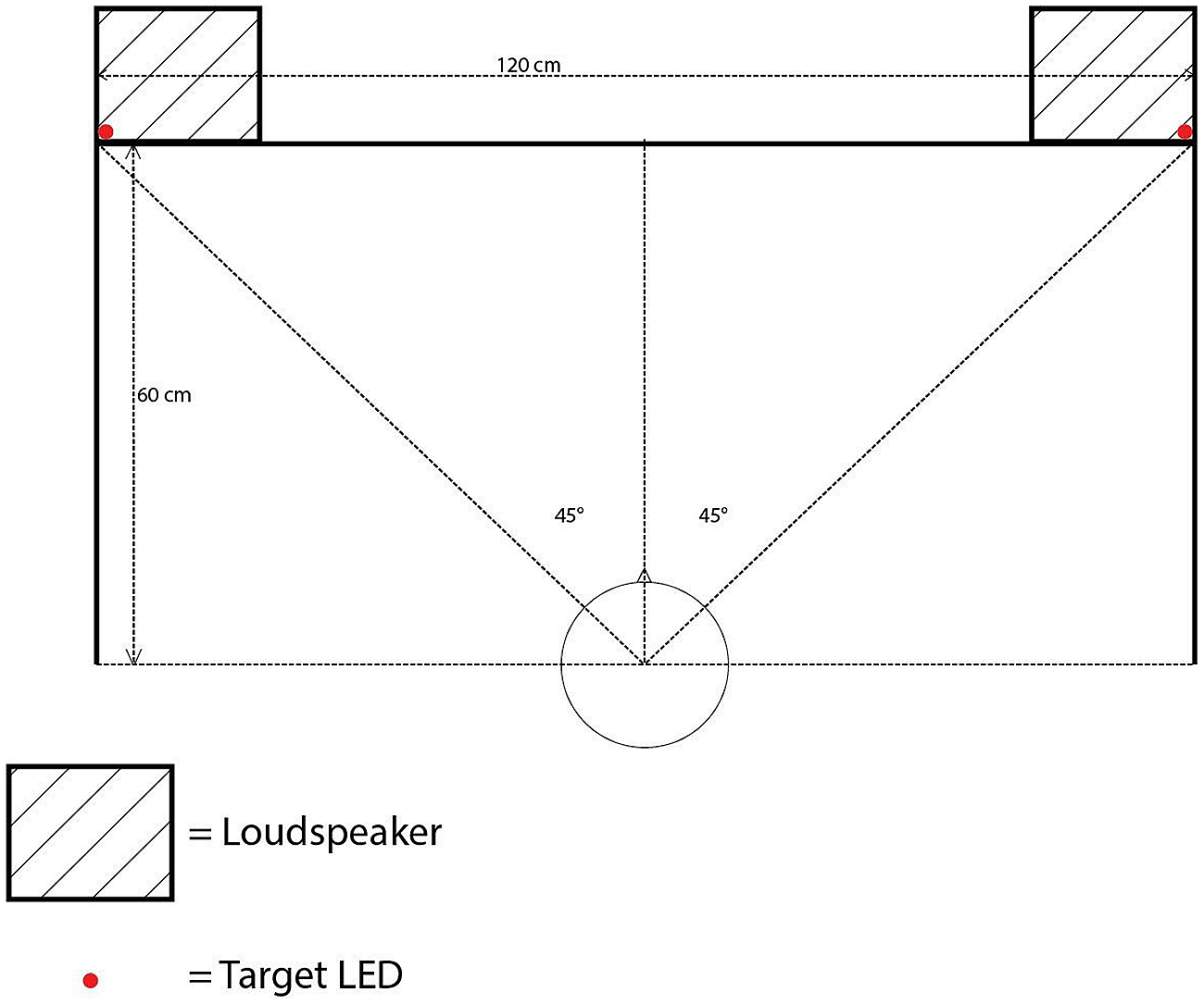
**Bird’s-eye view showing the position of the loudspeakers and target LEDs in Experiment 1**.

All of the experiments reported in the present study were conducted in a darkened room (320 cm × 144 cm × 220 cm), using MATLAB r2014a with Psychtoolbox 3.0.12 on Ubuntu 14.04 LTS. The participants were seated at a desk with a backlit computer keyboard, approximately 60 cm away from a cloth screen mounted on the front wall of the room. The cloth screen hid five 12v 5 mm LEDs with a luminance of 8000 millicandelas and two loudspeakers (*M*-Audio Studiophile AV 40; model 9900-65140-00). The LEDs were controlled by an Arduino Uno board rev. 3, following MATLAB commands. One LED was placed at the center of the screen, approximately at the eye level of the participants (111 cm from the floor) as a fixation point. Four additional LEDs were installed as visual targets in the top-left, top-right, bottom-left, and bottom-right positions, each separated by 60 cm horizontally and by 40 cm vertically with the fixation LED positioned in the center (see **Figure 1**).

The loudspeakers were equipped with a 1-inch diameter treble tweeter and a 4-inch diameter low frequency driver, positioned 10 cm below the tweeter. The loudspeaker frequency response ranged from 85 Hz to 20 kHz. The loudspeakers were placed on their sides so that the tweeters were situated closer to the walls than the low frequency drivers. The farthest sides of the two loudspeakers were separated by a distance of 120 cm. The center of each treble tweeter and low frequency driver was placed 111 cm above the floor. The auditory cues consisted of a 2000 Hz pure tone at 75 dBA and white noise (with a frequency cutoff range between 0 and 22 kHz) presented at 68 dBA, both measured from the participant’s ear position^2^. The sample rate for both auditory cues was 44.1 kHz. A computer monitor (Dell UltraSharp; model 1908FPb) was placed on the left side of the participant’s seat to display any instructions.

#### Design

There were three within-participants factors in the experiment: Cue Type (pure tone vs. white noise), Spatial Cuing (cue presented on the same vs. opposite side as the target), and stimulus onset asynchrony (SOA) between the cue and target (100, 200, or 700 ms). The crossing of these factors yielded 12 possible conditions, with each condition being presented 12 times randomly in each block of 144 trials. The participants completed a total of three blocks, and were encouraged to take a short break between blocks.

#### Procedure

At the start of each trial, the fixation LED was illuminated and remained on for 2 s after the onset of the visual target, or until the participant made a response. After a random delay of 400–650 ms, an auditory cue was presented from one of the two loudspeakers at a constant intensity, for 100 ms. A visual target, shown as the illumination of one of the four LEDs for 140 ms, occurred after a further delay of 0, 100, 600 ms, depending on the SOA. The participants were instructed to press the *up* arrow key on the keyboard if an LED illuminated on either the upper-left or upper-right, and to press the *down* arrow key if an LED illuminated on either the lower-left or lower-right. The participants were further instructed to ignore the auditory cue, and to respond as rapidly and accurately as possible to the location of the visual target. The participants completed 10 practice trials before the experimenter stepped out of the room. If the participants failed to respond within 2 s of the onset of the visual target, the trial terminated, and the next trial began.

### Results

A box plot of participants’ average RTs across all conditions revealed a median of 429 ms, between 354 and 524 ms for the 25-percentile (Q_1_) and 75-percentile (Q_3_) range, respectively. One participant’s average RT (*M* = 813 ms) was greater than the upper limit (Q_3_ + interquartile range multiplied by 1.5) when compared to that of the sample. This participant’s data was therefore identified as an outlier and removed from the analyses (see Tukey, 1977; Wesslein et al., 2014). The data from another participant were removed due to his/her failing to respond on more than 10% of all trials. The following trial data were excluded from the subsequent analyses: incorrect responses, responses immediately following an incorrect response, and RTs that fell outside the range between 150 and 1,500 ms (see Spence and Driver, 1997, for similar exclusion criteria). The application of these exclusion criteria led to the removal of a total of 404 trials (5.2% of the data).

**FIGURE 2:**
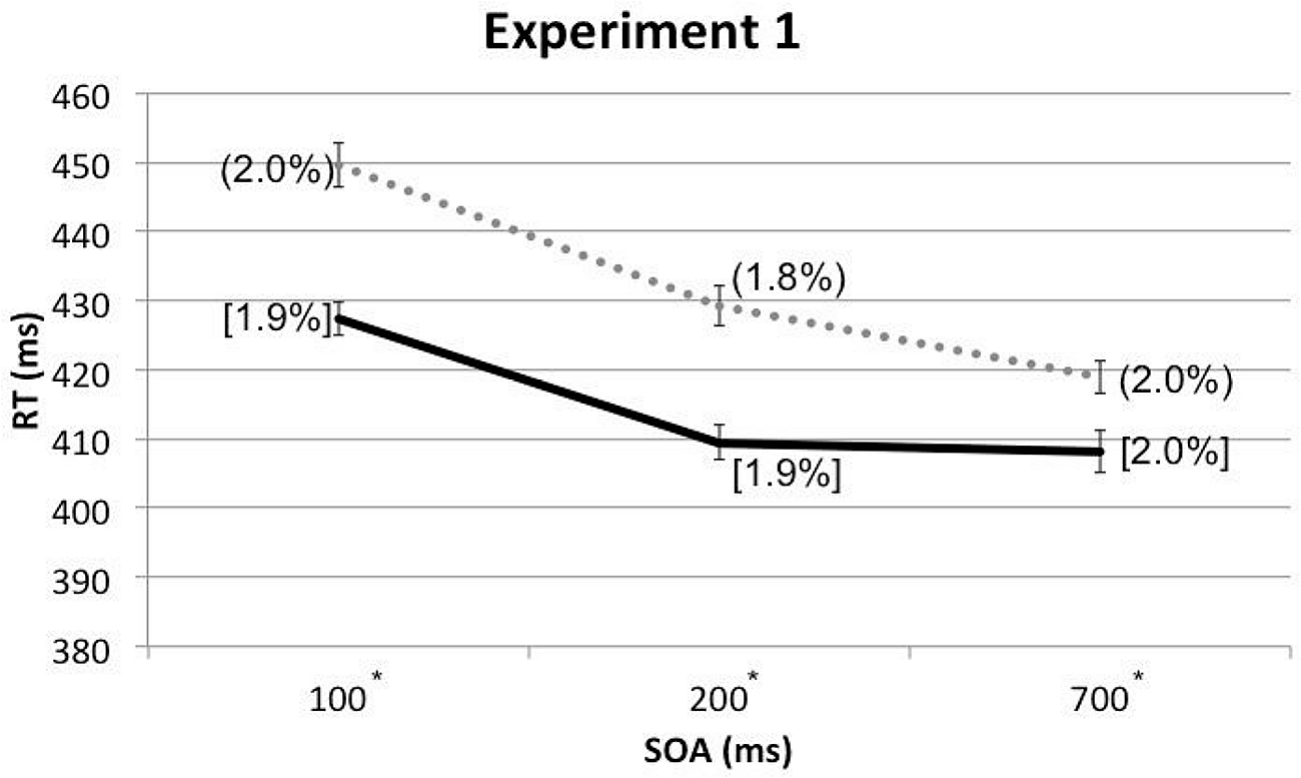
**Mean reaction times (RTs; in milliseconds) and error rates (ERs; cue-target cued conditions in square brackets and uncued conditions in rounded parentheses), as a function of cue-target stimulus onset asynchrony (SOA) in Experiment 1.** The solid line represents the cued conditions and the dotted line represents the uncued conditions. Asterisks indicate the RT differences between the cued and uncued conditions at given SOAs were significant based on paired *t*-tests after Bonferroni correction (*p* < 0.0167).

A three-way within-participants repeated measures analysis of variance (RM-ANOVA) was conducted with the factors of Cue Type, Spatial Cuing, and SOA. The analysis revealed a significant main effect of Spatial Cuing, *F*(1,17) = 47.489, *p* < 0.001, with participants responding more rapidly on the cued trials (*M* = 415 ms) than on the uncued trials (*M* = 433 ms). There was also a significant main effect of SOA, *F*(1.522,25.873) = 30.427, *p* < 0.001, with the participants responding more slowly at the 100 ms (*M* = 439 ms) as compared to either the 200 ms (*M* = 419 ms) or 700 ms (*M* = 414 ms) SOAs (the latter two conditions did not differ significantly, *p* = 0.384). This speeding-up of participants’ responses as the SOA increased presumably reflects a generalized alerting effect (see Spence and Driver, 1997). The analysis of the data also highlighted a significant two-way interaction between Spatial Cuing and SOA, *F*(2,34) = 5.935, *p* = 0.006. Paired *t*-tests revealed that the participants responded significantly more rapidly on the cued than on the uncued trials at all three SOAs: at the 100 ms SOA, *t*(17) = -6.575, *p* < 0.001; at the 200 ms SOA, *t*(17) = -6.159, *p* < 0.001; and at the 700 ms SOA, *t*(17) = -3.553, *p* = 0.002 (all *p*-values were smaller than 0.0167 based on Bonferroni correction, see **Figure 2** and **Table 1**). Subsequent contrasts revealed that the magnitudes of the cuing effects between the 100 ms (*M* = 22 ms) and 200 ms (*M* = 20 ms) SOAs were not significantly different, *F*(1,17) = 0.764, *p* = 0.394, whereas the magnitude of the cuing effect at the 200 ms SOA was significantly larger than that at the 700 ms (*M* = 11 ms) SOA, *F*(1,17) = 4.921, *p* = 0.040.

**Table 1:** Mean reaction times (RTs; in Milliseconds) from pure tone and white noise conditions, their within-participant SEs from Cousineau’s (2005) method, and error rates (ERs; in parentheses), as a function of stimulus onset asynchrony (SOA) and spatial cuing in Experiment 1.

		Pure tone		White noise	
SOA		*M*	SE	*M*	SE
100 ms	Cued	431 (1.6%)	3.2	424 (2.2%)	4.3
	Uncued	451 (2.0%)	5.1	448 (2.1%)	3.9
200 ms	Cued	408 (2.4%)	4.1	411 (1.4%)	4.2
	Uncued	430 (1.9%)	6.1	429 (1.7%)	5.1
700 ms	Cued	405 (2.0%)	4.2	411 (2.0%)	3.8
	Uncued	427 (2.2%)	5.0	411 (1.9%)	3.6

A similar analysis of the error data did not reveal any significant terms.

### Discussion

The results of Experiment 1 clearly demonstrate a significant exogenous crossmodal cuing effect. In particular, the participants’ elevation discrimination responses were facilitated when the presentation of the visual targets were preceded by an auditory cue on the same, rather than on the opposite, side of central fixation. These results therefore replicate those reported some years ago by Spence and Driver (1997; see Spence et al., 2004 for a review). However, another interesting result to emerge from the analysis of the data from our first experiment was that the magnitudes of the crossmodal cuing effects were similar regardless of the type of auditory cue (pure tone vs. white noise) that preceded the onset of the visual target. The latter result is interesting in that one might have expected, *a priori*, that more localizable auditory cues (i.e., the white noise burst) would have given rise to a more narrowly localized focusing of participants’ spatial attention around the cue location than the pure tone cues which were presumably less localizable in the elevation dimension (cf. Spence et al., 2004).

Having replicated the basic exogenous crossmodal spatial cuing effect and having demonstrated its seeming insensitivity to the type of auditory cue that was presented (at least for the two cues presented in Experiment 1), we went on, in Experiment 2, to investigate what would happen if the auditory cues were to be presented from behind the participant’s head on either the left or right (i.e., from a very different spatial location than that occupied by the visual target). The design of Experiment 2 was identical to that of Experiment 1, with the sole exception that on half of the trials, the auditory cues were now presented from behind the participant’s head, rather than from in front, in order to investigate whether they would also influence the speed of information processing for visual targets presented from the front. It was expected that the participants would respond more rapidly to the frontally arrayed visual targets after same side front cues (cued trials) than to the targets following front cues presented on the opposite side (uncued trials; thus hopefully replicating the results of Experiment 1). More interesting, though, was what would happen following the presentation of the same auditory cues from the rear. On the one hand, one might expect to observe no spatial cuing effects at all, since the rear cues would always be presented from a different *location* than the front targets. On the other hand, however, it could also be argued that the very fact that the cue and target are still presented on the same vs. opposite sides might be sufficient to elicit some sort of spatial cuing effect; who knows, perhaps the exact co-location of the cue and target would not matter. In fact, little is currently known about how attention is oriented exogenously following the presentation of auditory cues that fall outside of the visual field. Obtaining information on this point could be particularly interesting for those thinking about how to alert drivers, say, to stimuli presented in their blind spot (see Ho and Spence, 2008).

## Experiment 2

### Methods

#### Participants

Twenty-five participants (11 men and 14 women) took part in this study, recruited from the two sources used for Experiment 1 as well as from the Oxford University Experimental Psychology Research Participation Scheme. Nine of the participants had already taken part in Experiment 1. The mean age of the participants was 26 years, ranging from 20 to 41 years. There were 23 right-handed, 1 left-handed, and 1 ambidextrous participant by self-report.

#### Apparatus and Materials

**FIGURE 3:**
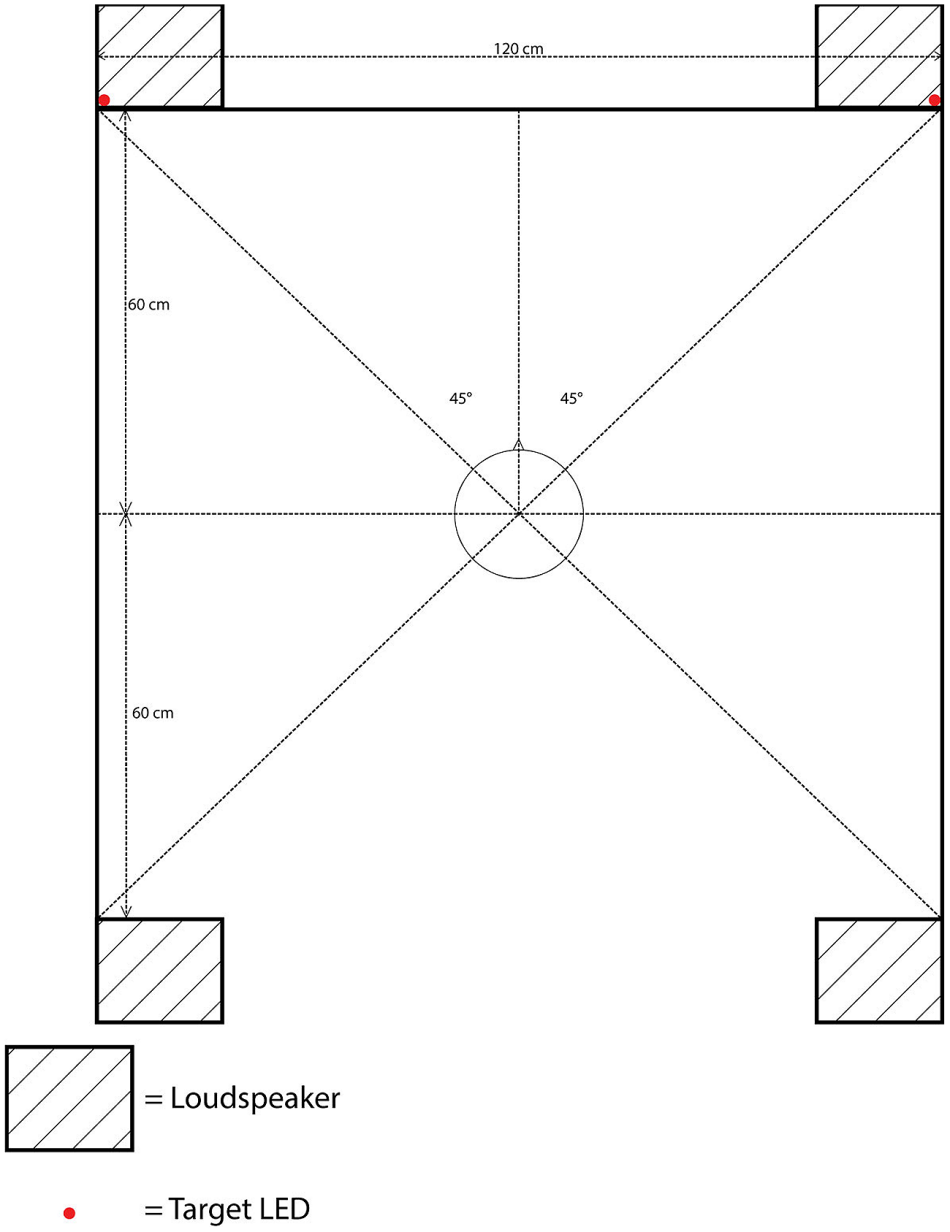
**Schematic bird’s eye view of the position of the loudspeakers, target LEDs, and the cloth screen in Experiment 2**.

The apparatus was identical to that used in Experiment 1, with the sole exception that an additional pair of loudspeakers was now placed behind the participant’s seat, parallel to the front loudspeakers. The distance between the front and rear loudspeakers was the same as that between the left and right loudspeakers: 120 cm, with each loudspeaker situated approximately 85 cm from the participants at 315° to front-left, 45° to front-right, 225° to rear-left, or 135° to rear-right position, with the central fixation LED located at 0° (see **Figure 3**).

#### Design and Procedure

This was exactly as for Experiment 1, with the sole exception that an additional within-participants factor, Cue Depth (front vs. rear), was also included in the experimental design. Hence, in each trial, an auditory cue would be presented from one of four locations: front-left, front-right, rear-left, or rear-right. This gave rise to a total of 24 possible conditions, with each condition occurring six times in a random order, once again giving rise to a total of 432 trials in three blocks.

### Results

The median of the participants’ averaged RTs across all conditions was 416 ms, ranging between 361 and 437 ms for the 25-percentile and 75-percentile, respectively. No outliers were identified, and all of the participants responded to more than 90% of the trials. A total of 351 trials (3.3%) were removed based on the exclusion criteria used in Experiment 1.

**FIGURE 4:**
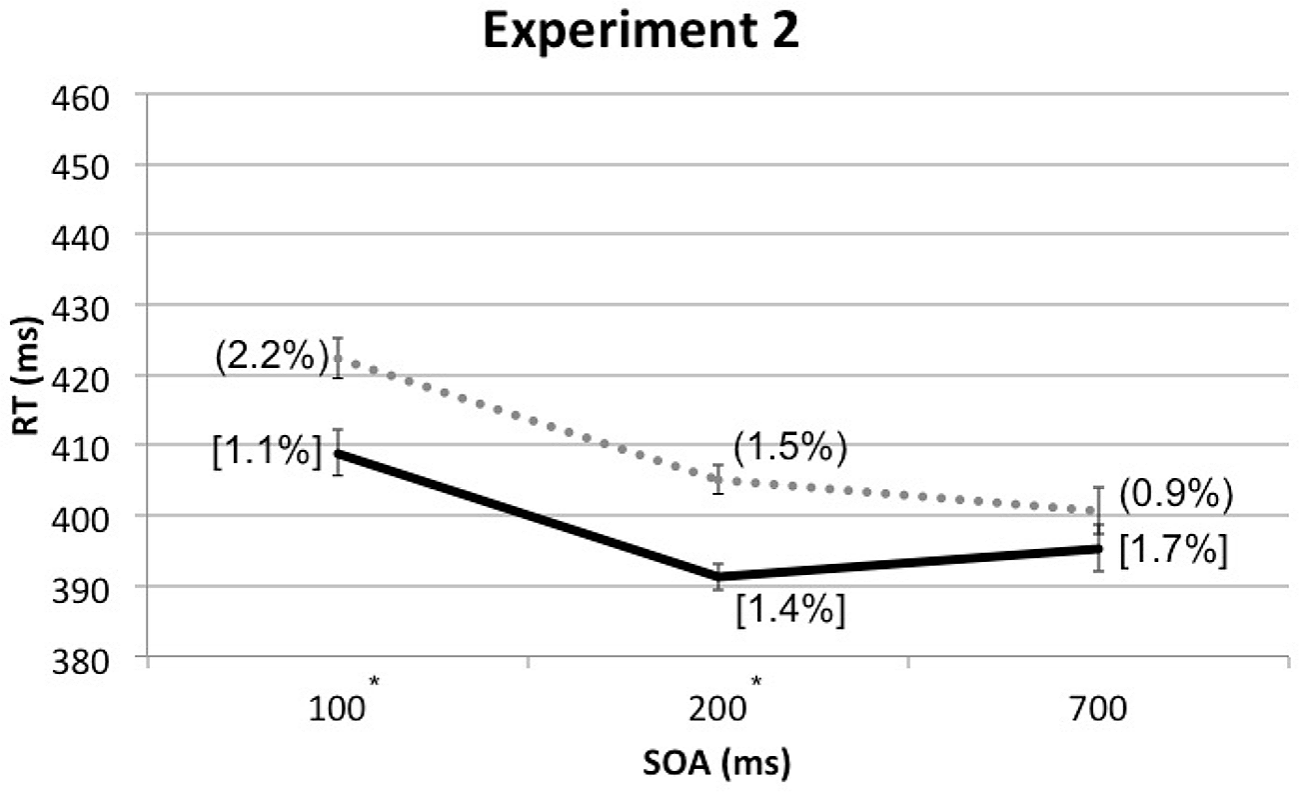
**Mean RTs (in milliseconds) in Experiment 2 as a function of cue-target SOA, with ERs for cue-target cued conditions in square brackets and ERs for uncued conditions in rounded parentheses.** The solid line represents the cued conditions and the dotted line represents the uncued conditions. Asterisks indicate significant cuing effects at given SOAs based on paired *t*-tests after Bonferroni correction (*p* < 0.0167).

A four-factor RM-ANOVA, which included the additional factor of Cue Depth (front vs. rear), along with the other three factors from Experiment 1, once again revealed a significant main effect of Spatial Cuing, *F*(1, 24) = 25.344, *p* < 0.001, with participants responding significantly more rapidly when the targets were presented from the same side as the cue than when the cue and target were presented from opposite sides. The analysis also revealed a significant main effect of SOA, *F*(1.225,29.400) = 16.064, *p* < 0.001. As in Experiment 1, the participants responded significantly more slowly to those targets presented at the shortest SOA (*M* = 416 ms) than at the other, longer, SOAs (*M* = 398 ms for both). A significant interaction was found between Cue Depth and SOA, *F*(2,48) = 4.674, *p* = 0.014, though paired *t*-tests revealed no significant differences between front and rear at any SOA: at 100 ms SOA, *t*(24) = 1.327, *p* = 0.197, at 200 ms SOA, *t*(24) = -1.831, *p* = 0.079, and at 700 ms SOA, *t*(24) = -2.037, *p* = 0.053. The two-way interaction between Spatial Cuing and SOA was marginally significant, *F*(2,48) = 3.043, *p* = 0.057. Paired *t*-tests revealed that the participants responded significantly more rapidly on the cued as compared to the uncued trials at both the 100 ms SOA, *t*(24) = -3.920, *p* = 0.001, as well as the 200 ms SOA, *t*(24) = -4.873, *p* < 0.001. However, the trend toward a cuing effect at the 700 ms SOA failed to reach significance, *t*(24) = -1.771, *p* = 0.089 (see **Figure 4** and **Table 2**).

**Table 2:** Mean RTs (in milliseconds) from front and rear conditions in pure tone and white noise conditions, their SEs based on Cousineau’s (2005) method, and ERs (in parentheses), as a function of SOA and spatial cuing in Experiment 2.

		Pure tone				White noise			
SOA		Front		Rear		Front		Rear	
		*M*	SE	*M*	SE	*M*	SE	*M*	SE
100 ms	Cued	413 (1.1%)	4.8	406 (1.6%)	3.5	410 (0.7%)	4.5	407 (0.9%)	5.5
	Uncued	423 (2.2%)	4.2	414 (2.2%)	3.2	424 (3.1%)	3.2	428 (1.3%)	5.3
200 ms	Cued	389 (0.9%)	3.2	395 (2.0%)	3.0	386 (1.6%)	3.9	394 (1.1%)	3.5
	Uncued	406 (1.8%)	4.8	406 (2.0%)	3.3	404 (0.4%)	3.9	404 (1.8%)	3.1
700 ms	Cued	397 (1.6%)	4.8	397 (1.3%)	4.7	394 (2.2%)	4.3	393 (1.8%)	4.8
	Uncued	401 (0.9%)	3.7	403 (1.3%)	4.3	392 (0.9%)	5.0	407 (0.4%)	4.8

Analysis of the error data revealed a significant two-way interaction between Spatial Cuing and SOA, *F*(2,48) = 7.041, *p* = 0.002. Pairwise comparisons (*t*-tests) revealed that the participants made significantly more errors on the uncued trials than on the cued trials at the 100 ms SOA, *t*(24) = -3.784, *p* = 0.001. Importantly, this pattern of results indicates that the faster responding observed in the former condition reflects the result of a genuine perceptual facilitation, not just some form of a speed-accuracy trade-off (cf. Spence and Driver, 1997).

### Discussion

The results of Experiment 2 once again replicate the basic crossmodal exogenous spatial cuing effect. For the second time in the present study, the nature of that cue (e.g., pure tone vs. white noise) had no impact on the pattern of results that was obtained. More interestingly, the magnitude of the crossmodal cuing effect was equivalent no matter whether the auditory cue happened to have been presented from in front of, or behind (i.e., outside the visual field of) the participants. Such a pattern of results can be taken to support the rather surprising conclusion that the exact co-location of auditory cues and visual targets is by no means necessary when it comes to eliciting a significant crossmodal spatial cuing effect. Instead, it would seem to be that the presentation of an auditory cue on the left or right side gives rise to a lateralized shift of visual attention regardless of the exact location from which that cue happens to be presented.

Here it is worth pausing for a moment to note how such a pattern of results provides a striking contrast from the within-hemifield modulation of crossmodal spatial cuing effects reported previously by Spence et al. (2004). Using eight possible visual targets (two *up* and two *down* targets on each side), Spence et al. (2004) reported that their participants’ RTs were faster when the visual targets were presented from the same lateral location (within the same hemifield) as the auditory cues than when the cues and targets were separated by 26°, 52°, or 78° laterally. In other words, the participants’ performance was better with visual targets that appeared from exactly the same lateral location as the auditory cues than with those trials where the target appeared from the same hemifield but at a different lateral location. So what might account for the difference in results between those reported by Spence et al. (2004) and those documented in Experiment 2 of the present study? Well, one intriguing possibility here relates to the fact that in the former study, all four potential auditory cue locations fell within the participant’s visual field. By contrast, here on half of the trials, the auditory cues were actually presented from a region of space for which the participant had no immediate visual representation (see Kennett and Driver, 2014).

One might also wonder about whether the size of the receptive fields in areas such as the superior colliculus that are known to be involved in such orienting responses might not be much broader in the far periphery than they are in the central visual field^3^ (see Rafal et al., 1991; Stein and Meredith, 1993). However, before such a conclusion can be accepted, it is important to rule out a potential alternative explanation for the pattern of results reported in Experiment 2. It is possible that the participants may simply have confused the perceived location of the auditory cues in the front–back dimension. Note here that both front and rear cues were located approximately 85 cm from the participant’s head, with the same azimuth (45°) from the participants’ ear positions (thus potentially falling in what is known as the *cone of confusion*, see Blauert, 1983; Butler, 1986; Makous and Middlebrooks, 1990; Middlebrooks and Green, 1991). Should the participants in Experiment 2 have experienced such confusion then this might provide an alternative explanation for the equal magnitude of cuing effects from the front and rear auditory cues. In order to evaluate the plausibility of this suggestion, we conducted a third and final experiment in which the ability of participants to correctly discriminate the location of the auditory stimuli was assessed.

## Experiment 3

### Methods

#### Participants

Twenty-five participants (11 men and 14 women) were recruited. Twenty-two of whom (10 men and 12 women) had taken part in Experiment 2. The mean of the participants’ ages was 25 years, ranging from 17 to 41 years. Twenty-three of the participants were right-handed, one was left-handed, and one was ambidextrous, by self-report.

#### Apparatus, Materials, Design, and Procedure

These were exactly as for Experiment 2, with the sole exception that the four visual target LEDs were not used in the present study. There were three within-participants factors: Target Type (pure tone vs. white noise), Target Depth (front vs. rear), and Target Side (left vs. right). The procedure was identical to that used in Experiment 2, with the sole exception that there were no visual targets. Instead, what had been auditory cues in Experiment 2 now became the auditory targets. They were presented following the illumination of the fixation LED with a random delay of between 400 and 650 ms. The participants simply had to respond to the location of the sound (now acting as the auditory target), pressing *7* for the sound from front-left, *9* for the front-right, *1* for the rear-left, or *3* for the rear-right sound. The participants were instructed to use both hands on the numeric keypad on the keyboard, and to respond as rapidly and accurately as possible. They were further instructed to ignore the sound type and to focus on the location of the sound. Importantly, no feedback on their performance was provided during the trials. Each participant completed a total of 144 trials in one block, with eight possible conditions randomly presented a total of 18 times.

### Results

The average ERs of participants were entered into a box plot, revealing a median of 8%, between 1 and 16% for the 25-percentile and 75-percentile range, respectively. One outlier (*M* = 52%) was removed from the data analyses based on Tukey’s (1977) method. All of the other participants responded on more than 90% of the trials.

The mean ERs of Target Side for the front and rear were entered into one-sample *t*-tests against 50% (i.e., the hypothetical performance ratio by chance) to assess if the participants could discriminate the auditory target locations between front and rear. The analysis revealed that the participants’ ERs at the front were significantly lower (*M* = 8%) than the ER by chance (*M* = 50%), *t*(23) = -20.216, *p* = 0.001. When targets were presented from the rear, the participants’ ERs (*M* = 12%) were significantly lower than chance (*M* = 50%), *t*(23) = -11.573, *p* = 0.001. These results show that the participants did not experience front–rear confusion. This suggests that the lateralized cuing effect from the rear auditory cues in Experiment 2 resulted from the crossmodal facilitation of the target processing by cues that could be presented from two very different locations on the same side.

A three-way within-participants ANOVA with the factors of Target Type, Target Depth, and Target Side revealed no significant main effects or interaction terms (see **Table 3**). It is noteworthy, however, that judgments of the side on which the sound was presented (left or right) were nearly perfect (>99% correct) when Target Depth and Target Side were analyzed separately, whereas judgments of Target Depth (i.e., front or rear) were not quite as good (mean accuracy of 90%).

**Table 3:** Mean ERs (in percentages) and their within-participant SEs based on Cousineau’s (2005) method from the pure tone and white noise conditions as a function of cue depth and cue side in Experiment 3.

		Pure tone		White noise	
Cue depth	Cue side	*M*	SE	*M*	SE
Front	Left	11.7%	3.6	12.1%	4.5
	Right	3.7%	2.8	4.9%	2.9
Rear	Left	14.1%	4.2	7.9%	3.1
	Right	14.1%	4.7	10%	4.8

A similar ANOVA on the RT data revealed a significant main effect of Target Side, *F*(1,23) = 7.117, *p* = 0.014, with participants responding significantly more rapidly to auditory targets on the right (*M* = 524 ms) than to those presented on the left (*M* = 550 ms), possibly reflecting the fact that the majority of the participants were right-handed. A borderline significant two-way interaction was found between Target Type and Target Depth, *F*(1,23) = 3.978, *p* = 0.058, with participants responding more rapidly to the pure tone targets (*M* = 535 ms) than to the white noise targets (*M* = 549 ms) in the front, but more slowly to pure tone targets (*M* = 551 ms) than to white noise targets (*M* = 513 ms) in the rear space. Paired *t*-tests revealed that, in rear space, the participants responded significantly more rapidly to white noise targets than to pure tone targets, *t*(23) = 2.851, *p* = 0.009.

### Discussion

The results of Experiment 3 demonstrate that the participants were not confused about the location from which the auditory stimuli were presented. As such, this result adds weight to the claim that crossmodal cuing effects lead to a lateralized shift of attention that can (at least under certain circumstances) be relatively insensitive to the exact co-location of the auditory cues and subsequently presented visual targets, at least when the discrepancy involves the presentation of auditory cues that fall outside of the visual field (cf. Spence et al., 2004). Before moving on, however, we thought it worthwhile to reanalyze the data from Experiment 2, given the fact that the majority of the participants took part in both Experiments 2 and 3 (i.e., 22 out of 25 participants). The participants were divided into two groups; specifically, those with high performance vs. those with low performance on the discrimination tasks for the auditory targets in Experiment 3. The data from the three participants who did not take part in Experiment 3 were removed from this analysis.

The RT data were divided into two groups based on the median ER in Experiment 3; the high Accuracy group consisted of those participants from Experiment 2 who made ≤8% errors in Experiment 3 (*N* = 13). The low Accuracy group consisted of the data from those participants who in Experiment 2 made >8% of errors in Experiment 3 (*N* = 9). The four within-participants factors (Cue Depth, Cue Type, Spatial Cuing, and SOA) were then entered into a RM-ANOVA, with the Accuracy group (high vs. low) as a between-participants factor. Significant interactions were obtained between Spatial Cuing and SOA, *F*(2,40) = 3.581, *p* = 0.037, and between Spatial Cuing, SOA, and Accuracy, *F*(2,40) = 4.297, *p* = 0.020.

**FIGURE 5:**
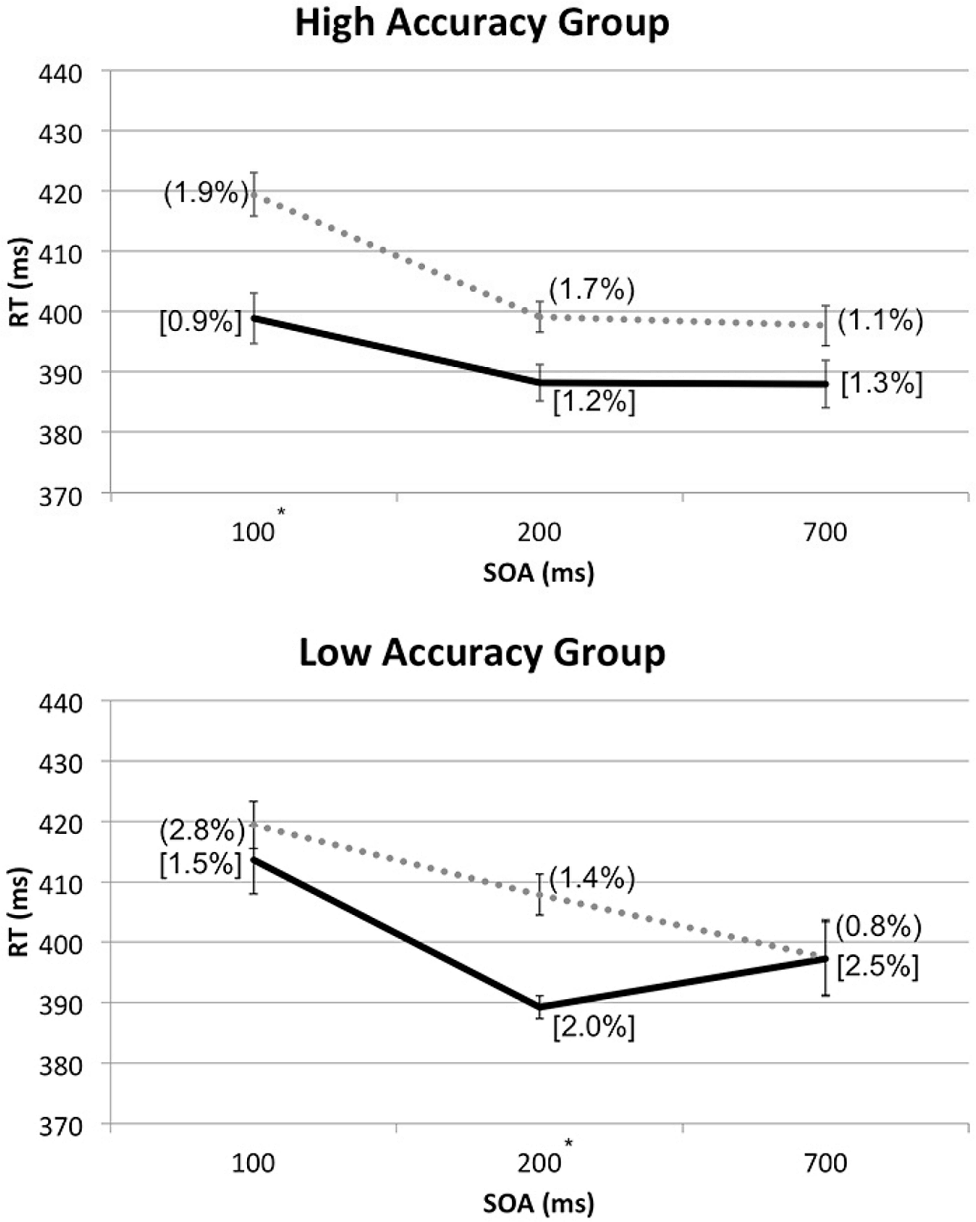
**Mean RTs (in milliseconds) from the reanalysis of the data from Experiment 2 shown as a function of cue-target SOA, divided into the high or low Accuracy groups based on the Experiment 3 sound localization performance.** For each group, ERs for cued conditions were shown in square brackets and those for uncued conditions entered in rounded parentheses. The solid line and dotted line are used to represent the cued and uncued conditions, respectively. Significant cuing effects were found at the SOAs with asterisks, based on paired *t*-tests with Bonferroni correction (*p* < 0.0167).

In order to break down the latter three-way interaction, additional RM-ANOVA tests with the factors of Spatial Cuing and SOA were conducted for the high and low Accuracy group separately. With the high Accuracy group, there was no significant interaction between Spatial Cuing and SOA, *F*(2,24) = 2.136, *p* = 0.140, meaning that the cuing effect was not affected by the SOAs. With the low Accuracy group, by contrast, a significant interaction was found between Spatial Cuing and SOA, *F*(2,16) = 7.066, *p* = 0.006. Paired *t*-tests revealed a significant cuing effect at the 200 ms SOA, *t*(8) = -5.294, *p* = 0.001 (see **Figure 5**).

Based on the lack of interaction between Spatial Cuing and SOA in the high Accuracy group, the crossmodal facilitation effect resulting from the presentation of the auditory cues that preceded visual targets on the same side, with the maximum benefit shown at the 100 ms SOA, seems to remain stable even at the 700 ms SOA for those who could easily discriminate the between left and right sounds as well as between the front and rear. The crossmodal facilitation effect for the low Accuracy group, on the other hand, seems to occur around 200 ms after the onset of the auditory cues preceding visual targets on the same side, and quickly disappears thereafter. In other words, the actual duration and timing of the facilitation effect for visual targets might vary depending on one’s accuracy in discriminating the locations of the auditory cue; the facilitation effect occurs sooner and lasts longer for those with high discrimination accuracy, as compared to those with low discrimination accuracy.

The results of this reanalysis of the data from Experiment 2 adds weight to the conclusion that, unlike the conventional belief (see Spence et al., 2004), the exact co-location of the auditory cue and the visual target is not always vital to elicit a significant crossmodal spatial cuing effect, at least not when the cue precedes the target on the same *lateral position, and outside the visual field*; the attentional shift elicited by an auditory cue, either at front or rear, will facilitate visual information processing speed for the frontal visual target on the same side.

A reviewer provided an alternative conclusion for these findings: in particular, s/he suggested that the observed lateralized spatial cuing effect may have been attributable to the difference in perceived loudness between the left and right side cues, regardless of whether the cues were presented from the front or rear. With those loudspeaker positions, the auditory cues on the same side, either from front or rear, fell on the cone of confusion, and therefore the front and rear cues on the same side had no distinguishable interaural differences not only in intensity but also in time (e.g., Röttger et al., 2007). If the participants could not distinguish front from rear, then their ERs at both front and rear should be 50%. Our aim in conducting Experiment 3 had been to address this possible alternative explanation. However, the results clearly indicate that there was no confusion in the front–back dimension; the participants could discriminate the cue locations between front and rear. It is possible that the participants solely relied on spectral cues by the reflection of the auditory stimuli in pinnae for the front–rear discrimination (see the head-related transfer function in Parseihian and Katz, 2012; Talagala et al., 2014). This would explain the higher ERs seen for Target Depth judgments than for Target Side judgments in Experiment 3; the loudspeaker set-up provided the participants three different types of spatial information to judge the location of cues between left and right, but only one to judge between front and rear.

## General Discussion

The results of the three experiments reported in the present study support a number of important conclusions concerning the crossmodal orienting of spatial attention (see Van der Stoep et al., 2015, for a review). First, the presentation of an auditory cue induced a robust short-lasting crossmodal exogenous cuing effect; this despite the fact that the auditory cue was entirely task-irrelevant throughout the present cuing studies. Importantly, the participants were able to discriminate the elevation of the visual targets more rapidly (and no less accurately) with the auditory cue on the same side as the target than when the cue was presented on the opposite side, regardless of the cue type.

These results confirm and extend the previous findings from unimodal (e.g., Posner and Cohen, 1984; Rafal et al., 1991; Spence and Driver, 1994) and crossmodal research (e.g., Spence and Driver, 1997) that the exogenous crossmodal facilitation effect is relatively short-lived; cuing effects were reliably detected only at the 100 and 200 ms SOAs. However, the reanalysis of the data from Experiment 2 suggests that the duration and possibly timing of crossmodal cuing effects may differ between those with high discrimination accuracy and those with low discrimination ability on auditory locations.

The most interesting yet surprising finding to emerge from the present study is that auditory cues from very *different spatial locations* on the same side of space can still facilitate the perception and discrimination of the elevation of the visual targets. According to the results reported here, all that seems to matter is that the cues and targets are presented from the same *side*. Given the fact that the distance between front and rear cue/target location was the same as the distance between left and right cue/target location (120 cm in both cases), the facilitation effect with rear cues cannot be explained by the proximity between rear auditory cues and frontal visual targets. Instead, it would appear that the relative side on which the auditory cue and visual target was presented (same vs. opposite) was the deciding factor for spatial cuing effect, not the exact co-location of the cue and target.

The lateralized shift of visual attention found in Experiment 2 can be understood in terms of the auditory system guiding visual attention; the auditory system detects the azimuth of the sound-emitting object in relation to one’s current position, and the motor and/or visual systems turn one’s eyes/head to the correct *side* in order to bring the object into vision accordingly (see Spence and Driver, 1997). In fact, it has been argued that the main purposes of the auditory system are to function as a warning system to help one prepare for motor behavior (Guski, 1992), and to compensate for the limited width of the visual field by guiding the eyes to that location for further visual evaluation (Heffner and Heffner, 1992; Heffner et al., 2001, 2007). Indeed, as Kubovy and Van Valkenburg (2001) put it more than a decade ago, the auditory system is “in the service” of visual system orienting toward the region of interest (see Arnott and Alain, 2011, for a review).

The results of the experiments reported here shows that, for the first time in research on crossmodal spatial cuing effects, the relative location of the cues and targets does not always matter. Here, equivalent cuing effects were elicited by both rear and frontal auditory cues prior to the presentation of frontal visual targets. Given the particular experimental settings used here, however, it remains unclear whether or not the absolute *lateral* location matters. According to the findings of Spence et al. (2004), it is possible that rear auditory cues at the same lateral locations as the frontal visual targets would have induced larger spatial cuing effects than those at different lateral locations from, yet still within the same hemifield as, the targets presented from the front.

## Conclusion

We would argue that the results of the present study may have important implications for the design of auditory warning signals in an applied setting. For example, modern vehicles come with auditory warning systems that detect nearby objects and generate collision alarm sounds. Unfortunately, to the best of our knowledge, the auditory warning systems are programmed to produce alarm sounds using all of the loudspeakers in the vehicles; currently we are unaware of any directional warning sounds being used. The warning sounds would perhaps be more useful if they *cue* the drivers’ attention to the object locations (see Ho and Spence, 2008). These results do, however, question whether it will be possible to direct a driver’s attention to the region of space that falls in their blind spot (see Ho and Spence, 2008). That said, auditory warning sounds can clearly still be effective in terms of eliciting a lateralized shift of a person’s attention even if their origin falls outside the current visual field of the driver.

## Conflict of Interest Statement

The authors declare that the research was conducted in the absence of any commercial or financial relationships that could be construed as a potential conflict of interest.

## Footnotes

1 Sometimes cuing effects reverse at longer intervals, a phenomenon known as Inhibition of Return, (see Posner and Cohen, 1984; Müller and Rabbitt, 1989; Klein, 2000), especially with those tasks that involve simple detection.

2 The two types of auditory cue were presented at different volumes due to the technical difficulties associated with matching them exactly.

3 Such a suggestion would certainly fit with evidence suggesting that vision serves to fine-tune auditory localization in central vision (Röder et al., 1999).
